# Genome and Bioinformatic Analysis of a HAdV-B14p1 Virus Isolated from a Baby with Pneumonia in Beijing, China

**DOI:** 10.1371/journal.pone.0060345

**Published:** 2013-03-29

**Authors:** Liuying Tang, Junjing An, Zhengde Xie, Shoaleh Dehghan, Donald Seto, Wenbo Xu, Yixin Ji

**Affiliations:** 1 National Institute for Viral Disease Control and Prevention, Chinese Center for Disease Control and Prevention, Beijing, People's Republic of China; 2 Fengtai District Center for Disease Control and Prevention, Beijing, People's Republic of China; 3 Beijing Institute for Pediatric Research, Capital Medical University Affiliated Beijing Children’s Hospital, Beijing, People's Republic of China; 4 School of Systems Biology, George Mason University, Manassas, Virginia, United States of America; 5 Chemistry Department, American University, Washington, D.C., United States of America; Columbia University, United States

## Abstract

The genome of HAdV-B14p1 strain BJ430, isolated from a six-month-old baby diagnosed with bronchial pneumonia at the Beijing Children’s Hospital in December 2010, was sequenced, analyzed, and compared with reference adenovirus genome sequences archived in GenBank. This genome is 34,762 bp in length, remarkably presenting 99.9% identity with the genome from HAdV14p1 strain 303600, which was isolated in the USA (2006). Even more remarkable, it is 99.7% identical with the HAdV-B14p (prototype “de Wit” strain) genome, isolated from The Netherlands in 1955. The patient and its parents presumably had no or limited contact with persons from the USA and Ireland, both of which reported outbreaks of the re-emergent virus HAdV-14p1 recently. These genome data, its analysis, and this report provide a reference for any additional HAdV-B14 outbreak in China and provide the basis for the development of adenovirus vaccines and molecular pathogen surveillance protocols in high-risk areas.

## Introduction

Human adenoviruses(HAdVs)are typed and ordered into seven species (A to G) with greater than 64 genome types reported in which the associations for specific human diseases are characterized [1], [2]. For example, HAdVs classified in species B, C, and E are known to cause respiratory infections [3]. Specifically, within species B, HAdV-B3, -B7, -B16, and -B21, belonging to subspecies B1, are respiratory tract pathogens, as are HAdV-B14, -B35 and -B55, which are members of subspecies B2.

HAdV-B14p was first isolated from The Netherlands and linked to a respiratory tract disease outbreak in military recruits between April and May, 1955[4]. This particular virus was associated with sporadic cases of acute respiratory disease (ARD) in Europe and Asia through the 1960s [4], [5] and then was not reported for a long period. In the approximately 50-year interval from the original identification to the recent outbreaks, reports of respiratory disease associated with HAdV-B14p were rare and limited to small numbers of patients [6]. Recently, from 2005 through 2009, HAdV14p1 has apparently re-emerged and has been associated with several large ARD outbreaks across the USA, associated with nine military and twenty-four civilian communities in contrast to the past, as well as in Europe (Ireland) [6]–[8]. Several HAdV-B14-like infections have also been recently reported in China, beginning from 2010 [9], [10]. The applications of genomics and bioinformatics to the adenoviral genomes provide high resolution insights into their epidemiology and evolution, specifically revealing the molecular basis for the genesis of several emergent and re-emergent adenovirus pathogens [2], [11]–[20]. This report presents the isolation and identification of a type 14 adenovirus, isolated from a six-month-old baby diagnosed with bronchial pneumonia. It was confirmed as HAdV-B14p1 by the analysis of its genome and, in particular, the hexon, fiber, and E1A genes. The genome was sequenced, analyzed, and compared with other reference adenovirus genome sequences archived in GenBank. This analysis shows that the Beijing HAdV14p1 has a close phylogenetic relationship with HAdV-B14p, isolated in 1955 from The Netherlands. Its sequence is remarkably conserved, given the time and geographic distances. It is also nearly identical to a HAdV-B14p1 strain (303600) recently characterized from an outbreak in the USA (2006). Given the recent outbreaks of this particular virus in the USA, Europe and China, these genome data and this report provide a reference for recognizing any future HAdV-B14 outbreak in China and serves as a foundation for the development of adenovirus vaccines and surveillance protocols in high-risk areas. Further, these observations will aid the continuing research and development of adenovirus-based vectors.

## Materials and Methods

### Virus recovery and DNA extraction

The specimen was isolated from a nasopharyngeal aspirate of a six-month-old infant diagnosed with bronchial pneumonia at the Beijing Children’s Hospital on December 2010. The sample collection and detection protocols were approved by the Ethics Review Committee from the National Institute for Viral Disease Control and Prevention, Chinese Center for Disease Control and Prevention. As such, the parents of the patient have given written informed consent to publish these case details. The sample was detected as adenovirus positive using a polymerase chain reaction (PCR) protocol. Subsequently, specific primers were designed for sequencing the open reading frames of the E1A, hexon, and fiber genes. Virus from this and other clinical samples were grown in Hep-2 cells; this sample presented a characteristic cytopathic effect (CPE), which was observed after 10 days of culturing. Viral genomic DNA was extracted from 140 µl of infected cell lysate using a QIAamp Viral RNA Mini kit (Qiagen, Ltd.; Germany), applied according to the manufacturer’s instructions.

### PCR amplification and sequencing strategy

Appropriate primers were designed using reference HAdV sequences that are available from GenBank. For genome DNA sequencing, a PCR strategy with 64 primer pairs was employed to amplify the the whole genome with overlapping fragments. The linear end-terminal sequences were determined using a procedure described previously. All of the reported sequences are the result of at least two sequencing reactions include both directions.

### DNA sequencing

Amplified genomic fragments were purified using agarose gel electrophoresis with a QIAquick gel extraction kit, as per the manufacturer’s instructions (Qiagen, Ltd.; Germany). Sanger-based DNA sequencing reactions were performed bi-directionally using an ABI Prism Big Dye Terminator v3.1 Cycle Sequencing Ready Reaction kit and AmpliTaq DNA polymerase (Applied Biosystems, Inc.; Foster City, CA). DNA sequencing ladders were resolved using an ABI 3730 DNA sequencer (Applied Biosystems, Inc.; Foster City, CA). Unresolved and ambiguous sequences were re-sequenced with additional custom primers located adjacent to the regions in question.

### Bioinformatic Analyses

The genome was assembled using Sequencher (v4.0.5; Gene Codes, Inc.; Ann Arbor, MI). For genome sequence annotation, the assembled genome was partitioned into 1-kb non-overlapping segments, similar to the method of Lauer et al, and each segment was systematically queried against the GenBank non-redundant sequences database using their BLASTX program. DNA sequence alignments were revealed using the BioEdit sequence alignment editor software (v5.0.9; T. Hall, North Carolina State University) and an online Gene-wise program (http://www.ebi.ac.uk/Wise2/advanced.html). All sequences were submitted for BLAST analysis (http://blast.ncbi.nlm.nih.gov/Blast.cgi/) to identify their closely related counterparts. As a quality control check, the annotated coding sequences corresponded to their counterparts from other HAdV genomes. Annotation of the splice positions and correlation with functional properties were completed by using an on-line splice prediction software (http://www.fruitfly.org/seq_tools/splice. html) and the GENSCAN software (http://genes.mit.edu/GENSCAN.html).

For genome and gene phylogenetic analysis, the forward and reverse sequence data were re-aligned and re-assembled into a single consensus sequence using the software MEGA v4.0 (Molecular Genetic Analysis Software; http://www.megasoftware.net). This confirmed the Sequencher-based assembly. Phylogenetic analysis was subsequently performed using MEGA as well, via the maximum-composite-likelihood method that generated neighbor-joining and bootstrapped trees of phylogeny with 1,000 replicates; all other parameters were set by default. Sequence percent identities were calculated using software which was part of the EMBOSS package (http://www.ebi.ac.uk/Tools/emboss/). Comparison of mutations across the genomes was done using unpublished software developed recently (D.S.).

GenBank archived sequences and accession numbers. The following genomes were used for these analyses, with details given for the type 14 strains (“p” denotes prototype; if no designation is noted, the strain is the prototype): HAdV-B14p1 USA/303600/2006 (FJ822614); HAdV-B14p1 China/BJ430/2010 (JN032132); HAdV-B14p Netherlands/de Wit/1955 (AY803294); HAdV-B55 China/QS-DLL/2006 (FJ643676); HAdV-B3 (AY599834); HAdV-B7 (AY594255); HAdV-B16 (AY601636); HAdV-B21 (AY601633); HAdV-B50 (AY737798); SAdV-21 (AC_000010); HAdV-B11 (NC_011202); HAdV-B34 (AY737797); HAdV-B35 (AY128640); HAdV-D9 (AJ854486); HAdV-E4 (AY594253); HAdV-G52 (DQ923122); HAdV-C1 (AF534906); HAdV-F40 (NC_001454); and HAdV-A12 (AC_000005).

Sequences used for the E1A analysis are as follows: HAdV-B11a USA/6380/1997 (FJ841919); HAdV-B11p USA/Slobitski/1956 (NC_011202); HAdV-B35p USA/Holden/1973 (AY128640); HAdV-B34p USA/Compton/1972 (AY737797); HAdV-B14p1 China/BJ430/2010 (JF438997); HAdV-B14p1 USA/303600/2006 (FJ822614); HAdV-B14p1 Ireland/2009 (HQ163916); HAdV-B14p1 USA/2971/2007 (FJ841915); HAdB-B55 China/QS-DLL/2006 (FJ643676); HAdV-B11a Taiwan/2474/2001 (FJ841922); HAdV-B11a Singapore/SNG1222/2005 (FJ841920); HAdV-B11a Taiwan/760/2002 (FJ841921); HAdV-B14p Netherlands/16845/1974 (FJ841917); HAdV-B14p Netherlands/deWit/1955 (FJ841918); and HAdV-B11a_Spain/273/1969 (FJ841916).

Hexon sequences used are as follows: HAdV-B11a USA/6380/1997 (FJ841899); HAdV-B11p USA/Slobitski/1956 (NC_011202); HAdV-B35p USA/Holden/1973 (AY128640); HAdV-B34p USA/Compton/1972 (AY737797); HAdV-B14p1 China/BJ430/2010 (JF420883); HAdV-B14p1 USA/303600/2006 (FJ822614); HAdV-B14p1 Ireland/2009 (HQ265808); HAdV-B14p1 USA/2971/2007 (FJ841901); HAdB-B55 China/QS-DLL/2006 (DQ874353); HAdV-B11a Taiwan/2474/2001 (FJ841906); HAdV-B11a Singapore/SNG1222/2005 (FJ841904); HAdV-B11a Taiwan/760/2002 (FJ841905); HAdV-B14p Netherlands/16845/1974 (FJ841902); HAdV-B14p Netherlands/deWit/1955 (AY803294); and HAdV-B11a Spain/273/1969 (FJ841900).

For the analysis of the fiber gene, these sequences were applied: HAdV-B11a USA/6380/1997 (FJ841907); HAdV-B11p USA/Slobitski/1956 (NC_01120); HAdV-B35p USA/Holden/1973 (AY128640); HAdV-B34p USA/Compton/1972 (AY737797); HAdV-B14p1 China/BJ430/2010 (JF420882); HAdV-B14p1 USA/303600/2006 (FJ822614); HAdV-B14p1_Ireland/2009 (HQ163916); HAdV-B14p1 USA/2971/2007 (FJ841909); HAdB-B55 China/QS-DLL/2006 (FJ643676); HAdV-B11a Taiwan/2474/2001 (FJ841914); HAdV-B11a Singapore/SNG1222/2005 (FJ841912); HAdV-B11a Taiwan/760/2002 (FJ841913); HAdV-B14p Netherlands/16845/1974 (FJ841910); HAdV-B14p Netherlands/deWit/1955 (AY803294); and HAdV-B11a Spain/273/1969 (FJ841908).

### Retrospective clinical investigation

Clinical records and laboratory test data were reviewed for a total of 44 patients who were hospitalized from 1 May through 31 August, 2010. These patients were housed in the same ward as the patient from which this HAdV-B14p1 BJ430 strain was isolated. The hospital staff collected 44 nasal pharynx aspirates, which were examined using PCR protocols. In these assays, viral pathogens known to cause acute respiratory disease, such as adenovirus, influenza, parainfluenza, SARS coronavirus, rhinovirus, and RSV, were screened using appropriate PCR primer sets.

## Results

### Comparative genomics of HAdV-B14p and two HAdV-B14p1 strains

**Figure 1:**
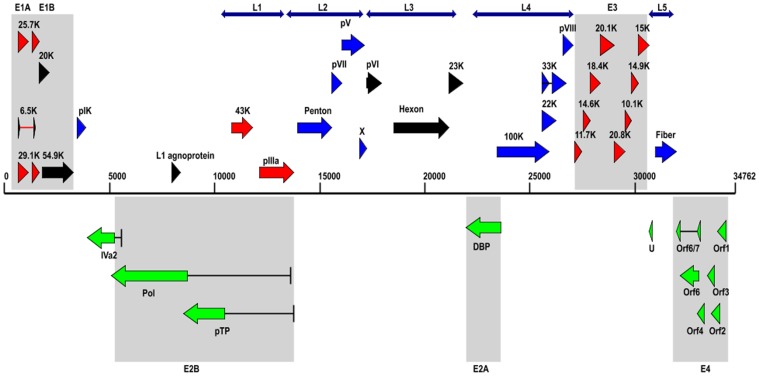
Genome organization of HAdV-B14p1 coding sequences. The genome is represented by a central black horizontal line marked at 5-kbp intervals. Protein encoding regions are shown as arrows indicating transcriptional orientation. Forward arrows (above the horizontal black line) show coding regions in the 5’ to 3’ direction and arrows pointing to the left (below the horizontal black line) show the coding regions encoded on the complementary strand. Spliced regions are indicated by a black line joining the coding sequences. Colors are added for contrast between the groups and not indicative of other relationships other than grouping the genes to their transcript, for example, the two red genes of “L1” have no relationships to the eight red genes of “E3”.

The complete genome of HAdV-B14p1 strain BJ430 contains 34,762 base pairs (bp). This is very similar to the genomes of the prototype and another HAdV-B14p1 virus (strain 303600; Lackland Air Force Base, USA), with genome sizes of 34,764 bp. Forty-two putative coding regions (Table 1) were identified in this genome along with conserved non-coding sequence motifs that are in common with HAdV-B14p1 (strain 303600) and the prototype (Table 2). A map of the genome organization of coding sequences from strain BJ430 is shown in Fig. 1. Note that the colors of the arrows are used for contrast only and to group the coding regions to the gene transcripts, e.g., E1A, L1, E2B, etc. The colors do not reflect any other relationships other than grouping the genes to their transcript, for example, the two red genes of “L1” have no relationships to the eight red genes of “E3”.

**Table 1:** Annotation of coding sequences from the genome of HAdV-B14p1 strain BJ430.

Gene	Product	Location
E1A	29.1 k Da protein	586–1165, 1250–1458
E1A	25.7 kDa protein	586–1072, 1250–1458
E1A	6.5 kDa protein	586–660, 1250–1354
E1B	20 kDa protein	1628–2170
E1B	54.9 kDa protein	1933–3417
pIX	pIX protein	3500–3919
IVa2	IVa2 protein	3987–5320, 5596–5608 (-)
E2B	DNA polymerase	5087–8659, 13644–13652
Hypothetical protein	L1 agnoprotein	7865–8275
E2B	pTP protein	8458–10419, 13644–13652
VA-RNA I	VA-RNA I	10451–10612
L1	43 kDa protein	10669–11829
L1	protein IIIa precursor	11855–13618
L2	penton protein	13701–15377
L2	protein VII	15382–15960
L2	protein V precursor	16003–17058
L2	protein X	17087–17317
L3	protein VI	17398–18138
L3	hexon protein	18254–21091
L3	23 kDa protein	21128–21757
E2A	DNA binding protein	21835–23391 (-)
L4	100 kDa hexon-assembly associated protein	23422–25860
L4	22 kDa protein	25592–26167
L4	33 kDa protein	25592–25910, 26080–26441
L4	protein VIII	26491–27174
E3	11.7 kDa protein	27174–27491
E3	14.6 kDa protein	27445–27840
E3	18.4 kDa protein	27825–28325
E3	20.1 kDa protein	28345–28890
E3	20.8 kDa protein	28908–29459
E3	10.1 kDa protein	29502–29777
E3	14.9 kDa protein	29782–30186
E3	15 kDa protein	30179–30586
U	U protein	30610–30774 (-)
L5	fiber protein	30789–31760
E4	Orf6/7 protein	31796–32047, 32770–32943 (-)
E4	Orf6 protein	32044–32943(-)
E4	Orf4 protein	32846–33214(-)
E4	Orf3 protein	33223–33576(-)
E4	Orf2 protein	33573–33962(-)
E4	Orf1 protein	34005–34382(-)

Nucleotide locations indicate the start and stop codons, with coding sequences transcribed from the complementary strand designated by “(-)”, e.g., “5596–5608 (-)”. Coding sequences with spliced regions are indicated by double entries in the location column.

**Table 2:** Comparison of non-coding sequence motifs between HAdV-B14p1 strains BJ430 and 303600.

MOTIF	function	BJ430	303600	bp
CATCAT...TGACGT	inverted terminal repeat	1–136	1–136	136
ATAATATACC	DNApol-pTP bingding site	9–18	9–18	10
TGGAATGGTGCCAA	NFI bingding site	26–39	26–39	14
ctgtgtgg	Sp1 recognition site	72–79	72–79	8
TATTTA	TATA signal for E1A	493–498	493–498	6
AATAAA	polyA signal for E1A	1517–1522	1517–1522	6
TATATA	TATA signal for E1B	1574–1579	1574–1579	6
AGTAAA	polyA signal for E1B	3467–3472	3467–3472	6
TAAGGT	TATA signal for IX	3415–3420	3415–3420	6
AAAAAT	polyA signal for IX	3470–3475	3470–3475	6
TTGATT	polyA signal for IVa2	3963–3968	3963–3968	6
TGATTGGCTT	inverted CAAT box for MLP	5858–5867	5858–5867	10
TTCACGTGA	upstream element for MLP	5877–5885	5878–5886	9
GCTGGGGGGG	MAZ bingding site for MLP	5898–5907	5899–5908	10
TATAAAA	TATA signal for major late promoter(MLP)	5908–5914	5909–5915	7
GGGGGCGGTTC	MAZ/SP1 bingding site for MLP	5915–5925	5916–5926	11
TCACTGT	initiator element for MLP	5937–5943	5938–5944	7
TTGTCAGTTTC	DE1 for MLP	6024–6034	6025–6035	11
AACGAGGAGGATTTGA	DE2a & DE2b for MLP	6039–6054	6040–6055	16
AATAAA	polyA signal for L1	13626–13631	13624–13629	6
AATAAA	polyA signal for L2	17337–17342	17335–17340	6
AATAAA	polyA signal for L3	21781–21786	21780–21785	6
AATAAA	polyA signal for E2A	21793–21798(-)	21792–21797(-)	6
TATAAA	TATA box for E3	26856–26861	26855–26860	6
AATAAA	polyA signal for L4	27335–27340	27334–27339	6
AATAAA	polyA signal for E3	30597–30602	30597–30602	6
AATAAA	polyA signal for L5	31763–31768	31763–31768	6
AATAAA	polyA signal for E4	31779–31784	31779–31784	6
TATATATA	TATA box for E4	34453–34460	34454–34461	8
AACCTC…ATGATG	inverted terminal repeat	34626–34762	34627–34763	137

Nucleotide signatures and putative functions are indicated, along with locations noted for the genome.

When the genome of the HAdV-B14p1 strain BJ430 was compared to those from the HAdV-B14p1 strain 303600 and the prototype HAdV-B14p “de Wit” viruses, the percent nucleotide identities were 99.9% and 99.7%, respectively (GenBank acc. no. FJ822614 and AY803294). However, there were sequence differences amongst them, indicating that although the HAdV-B14p1 viruses may have a common ancestry, they have diverged from one another recently.

Between the two genomes of HAdV-B14p1, and using BJ430 as the reference, there are eleven base substitutions, four single base insertions and two deletions, with one involving a single base (A) and the other involving TT. On the other hand, strain BJ430 differs from HAdV-B14p by 94 base substitutions and, relative to strain BJ430, there are five insertions (T, AAA, A, T, AGAAAA) and six deletions (GTG, T, TT, A, A, AA). Of these, the six-nucleotide deletion results in the deletion of amino acids 251 and 252 (Lys and Glu) that are located in the fiber knob near the recognized putative receptor-binding site [21] . In regards to the fiber gene, the sequence from strain BJ430 was identical to strain 303600. For the hexon gene, there was one single base substitution (G to A), which resulted in a synonymous mutation.

### Computational analysis of proteins

Presented in Table 3 are the percent identities of select proteins spanning the genome. Proteins from all prototype genomes from all HAdV species are represented, with one from each but including all subspecies B1 and B2 prototypes. One chimpanzee adenovirus (SAdV-B21) is included, as it segregates into the subspecies B1 subclade.

**Table 3:** Percent identities of select HAdV-14p1 strain BJ430 proteins with representative HAdVs from all species and including all species B viruses.

Protein	E1A 29.1-kDa protein	E1B 20-kDa protein	DNA polymerase	pTP	L1 43-kDa protein	L2 penton	L3 hexon	pVIII	L5 fiber	E4 34-kDa protein
HAdV-12 (A)	44	44	70	76	75	72	77	77	31	53
HAdV-11 (B2)	97	98	99	99	99	97	92	99	92	99
HAdV-14 (B2)	98	100	99	99	100	99	99	100	99	100
HAdV-34(B2)	98	99	99	99	99	95	94	99	63	98
HAdV-35 (B2)	96	98	99	99	95	97	91	99	62	98
HAdV-3(B1)	77	88	90	93	98	85	85	94	57	98
HAdV-7 (B1)	78	89	90	93	92	85	86	94	91	97
HAdV-16(B1)	78	90	91	94	92	85	85	94	51	89
HAdV-21 (B1)	79	90	91	93	92	91	90	94	62	97
HAdV-50 (B1)	78	90	91	93	92	91	93	94	61	97
SAdV-21 (B1)	80	88	92	93	92	90	93	95	55	86
HAdV-1(C)	36	49	76	78	82	69	76	80	29	62
HAdV-9 (D)	41	54	80	79	80	77	82	80	29	69
HAdV-4 (E)	56	59	84	88	90	82	82	89	28	70
HAdV-40 (F)	37	47	70	74	78	72	78	79	35	47
HAdV-52 (G)	36	42	76	78	82	72	79	78	28	51

The proteins span the entire genome.

### Phylogenetic analyses

**Figure 2:**
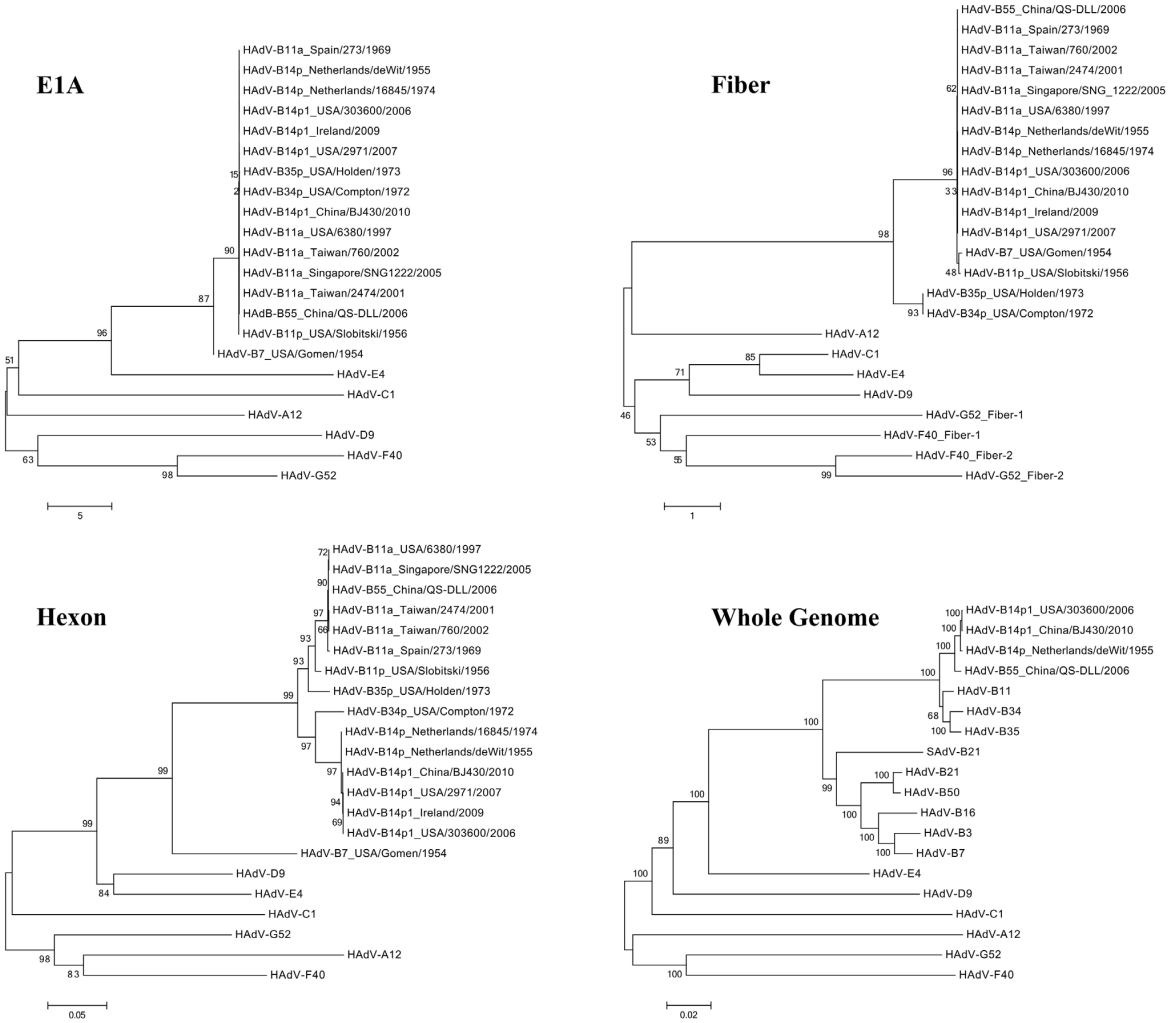
Phylogenetic analysis. E1A, fiber and hexon genes, as well as whole genome sequences of HAdV, are analyzed with respect to their phylogenetic relationships. Genes from the three recent HAdV-B14p1 strains are closely related to each other and to the prototype HAdV-B14p genome. It is remarkable that HAdV-B14p1 has a high level of sequence similarity to the prototype genome after approximately 50 years. Phylogenetic analysis was performed using the software MEGA v4.0 (Molecular Genetic Analysis Software; http://www.megasoftware.net), specifically applying a maximum-composite-likelihood method that generated neighbor-joining and bootstrapped trees of phylogeny with 1,000 replicates; all other parameters were set by default.

A whole genome phlyogenetic analysis of strain BJ430 was carried out in the context of all of the species B genomes along with a representative genome from each of the other six HAdV species (Fig. 2). This provides an understanding of the sequence relationships of this respiratory pathogen to other pathogens. HAdV-B14p1 strain BJ430 forms a subclade with other members of subspecies B2. This branched with a clade of all subspecies B1 genomes, and both form a clade that is distinct from the clades representing the other species. With bootstrap values greater than 80, there is high confidence in these phylogenetic relationships.

Three genes were also analyzed using phylogenetics(Fig. 2). These three genes (E1A, hexon, and fiber) were selected based on the GenBank availability of their sequences from viruses isolated in the past and associated with respiratory disease. Unfortunately whole genome data were not available for these viruses. Given the expense of DNA sequencing in the past, this was expected. Note: “HAdV-B11a” is the old name given to a respiratory pathogen that has been since re-named “HAdV-B55” based on its genome data and pathogenicity profile[22]. It is noted here as the serotype-based name “HAdV-B11a” to be consistent with the GenBank entries. E1A, hexon and fiber genes from both strains of HAdV-14p1, and also from another recent HAdV-B14p1 isolate (Ireland), form subclades with their counterparts from HAdV-14p. No whole genome data for the Ireland HAdV-B14p1 has been published.

## Discussion

In China, adenovirus is not recognized presently as a statutory infectious disease agent; therefore, reports of these viruses have not been incorporated into the National Infectious Disease Agent Monitoring System. Recently, adenovirus-linked respiratory infections are increasingly being reported in China, with multiple HAdV types identified as causative agents: HAdV-C1, -C2, -E4, -C6, -B3, -B7, -B11, -B14, and -B55 [9], [14], [22]–[25]. Presumably the “HAdV-B11” is a mischaracterization due to an incomplete assessment of the genome, that is, the fiber gene that may identify it as a HAdV-B55 virus was not assessed [22]. It should be noted that ‘true’ adenovirus type 11 viruses are renal tract pathogens, i.e., HAdV-B11p, -B11b, and –B11c. Whether this increase in adenovirus-linked respiratory diseases is due to better clinical detection and reporting or due to social events, e.g., global travel and interactions, is a matter of great concern to the public health of China. Improved detection, identification, and high resolution analysis of the genomes of these highly contagious emergent and re-emergent pathogens will enable a better understanding of this virus, and will provide a basis for enacting measures to prevent or limit its effect on the population. Here we report the details of the analysis of a HAdV-B14p1 genome recovered from a respiratory infection in a six-month-old baby from Beijing. It is the first bioinformatics report of a HAdV-B14 virus in China. This pathogenic agent was confirmed using virus isolation and with both molecular detection and serology.

HAdV-B14p1 has re-emerged after an approximately 50-year absence globally; it was linked to several ARD outbreaks in the Americas (USA), Europe (Ireland), and Asia (China), with reports of virus isolation and identification from 2007–2010. This virus is of great concern as it is highly contagious, with morbidity and mortality reported in the USA and Ireland outbreaks. In China, to date, the only reported cases have been sporadic and limited to single or a few patients. Further, all of the reported cases in China presented with mild to moderate clinical symptoms, without fatalities.

Access to the high resolution data embedded in the genomes provides the tools for understanding the evolution of the pathogen as well as insights into origins of new strains, dissemination, and epidemiology. The nucleic acids alignment of the HAdV-B14p1 genomes, along with three genes (hexon, fiber, and E1A) from these three widely separated global regions showed high levels of identity, suggesting a common ancestry. Sequence analysis shows a small number of genome changes between the recent China and USA strains of HAdV-B14p1, with nearly identical differences in both that are divergent from the genome sequenced from the prototype virus isolated in The Netherlands in 1955. These differences include a two amino acid deletion in the fiber gene, which suggests a shared lineage of these two HAdV-B11p1 viruses. Phylogenetic analysis of the three genes support the partition of HAdVs into species, originally based on biological, epidemiological, and structural attributes. Of particular note are the subclades of subspecies B1 and B2, which are closely related viruses. Of these, HAdV-B14 and -B55 are recent re-emergent respiratory pathogens; HAdV-B3, -B7, and -B16 are long-standing respiratory pathogens. As HAdV-B55 is a recombinant, involving a renal pathogen and a respiratory pathogen, and as HAdVs from species D HAdVs comprise prototypes of recombinogenic origins[26], clinicians and epidemiologists should factor this into surveillance and diagnostic protocols.

Phylogenetic analysis with available counterpart genes from the recent Europe (Ireland) isolate suggests it too is of the same lineage. This is particularly interesting, given no evidence of an epidemiologic connection for the strains from these different and widely separated countries. Regarding strain BJ430, the patient's parents are temporary workers in Beijing, originally from Nanchang (Jiangxi Province); they have no history of travel abroad and have limited if any interactions with foreigners. An epidemiological investigation was not able to determine the origins of the child's HAdV-B14p1 viral infection.

Presumably HAdV-B14p1 is transmitted from person to person, with the virus evolving and “adapting” in each round of replication [27]. New strains of HAdVs are problematic if there is no herd immunity present to counter that specific virus. Of additional concern is whether it may recombine with another HAdV to evolve into a more deadly or more contagious pathogen. An example of this is HAdV-B55, which has emerged and has been reported in China recently. The isolate QS-DLL was originally reported as “HAdV-B11a”, based on its hexon and fiber genes, but is recognized as a new genome type HAdV-B55 due to its whole genome identity to HAdV-B14 as well as its pathogenecity profile as a respiratory rather than a renal tract pathogen. HAdV-B55 is a recombinant adenovirus comprising a large portion of the HAdV-B14 genome and a much smaller portion of the HAdV-B11 genome. It is also an emergent pathogen in China as HAdV-B55 was identified as the virus responsible for a large respiratory disease outbreak in 2006, which included one fatality, and has been found subsequently to cause respiratory disease outbreaks in several provinces. It is a serious public health issue in both military and civilian populations nationally in China (unpublished). HAdV-B14p1 is not a recombinant genome as its genome is nearly identical to that of the prototype, which was examined for recombination events [28]. This recombinant analysis was repeated and confirmed for this study, given the influx of additional genomes into GenBank. The phylogenetic survey and protein percent identity analysis support this view as well. This particular type 14 genome data will enable the identification and further understanding of any future emergent recombinant HAdV resulting from adenovirus type 14.

To determine the source of these infections and to assess if there is nosocomial transmission, archived samples were examined under the auspices of an annual surveillance program for respiratory tract diseases at the Beijing Children's Hospital. The data set was collected from in-house patients during 2010. This retrospective study identified seven samples positive for HAdV (two were HAdV-C2; one was HAdV-C5; two were HAdV-B3; one was HAdV-B7; and one was HAdV-B14p1, described here). In addition, 20 samples were positive for RSV; five for parainfluenza viruses (four were type 3 and one was type 1); and eight were positive for rhinovirus. These included co-infections noted by PCR assays (unpublished data). The six-month-old infant presented in this report showed no co-infection with other respiratory viruses, including other types of adenovirus; therefore, there is no evidence of nosocomial transmission of this re-emergent adenovirus type 14.

Both epidemiological and virus surveillance of this previously unknown respiratory disease pathogen should be enhanced to provide more data for further understanding of its impact on the population, especially as a foundation for strategies to limit their impact on the population.

GenBank deposition. The genome and gene nucleotide sequences, along with the annotations of “Human adenovirus B human/CHN/BJ430/2010/14[P14H14F14]”, in the naming format preferred by the National Center for Biotechnology Information (NCBI) [29] were deposited in GenBank. These are available under the following accession numbers: JN032132 (genome); JF420883 (hexon); JF420882 (fiber); and JF438997 (E1A).
